# Abnormal Fetal Echocardiography in Diabetic Pregnant Women at a Tertiary Care Hospital: A Descriptive Cross-sectional Study

**DOI:** 10.31729/jnma.5178

**Published:** 2020-07-31

**Authors:** Jyotshna Sharma, Sanjeeb Tiwari

**Affiliations:** 1 Department of Obstetrics and Gynaecology, Kathmandu Medical College, Sinamangal, Kathmandu, Nepal; 2 Department of General Practice and Emergency Medicine, Maharajgunj Medical Campus, Institute of Medicine, T.U., Maharajgunj, Kathmandu, Nepal

**Keywords:** *diabetes mellitus*, *echocardiography*, *gestational diabetes*

## Abstract

**Introduction:**

The sedentary lifestyle of women and change in their food habits has a significant role in developing diabetes in pregnancies. This leads to an increased chance of fetal cardiac abnormality born by a mother with gestational diabetes and pre-existing diabetes. The objective of the study is to find out the prevalence of abnormal fetal echocardiography in gestational and pre-existing diabetic pregnant women at a tertiary care hospital.

**Methods:**

A descriptive cross-sectional study was conducted among 104 diabetic pregnant women in a tertiary care hospital from April 15, 2017, to April 14, 2018. Ethical approval was obtained from the institutional review committee. The convenient sampling method was used. The patients who were diagnosed as gestational diabetes and diabetic before pregnancy were included in the study. Fetal echocardiography was mainly done at a gestational age of 22-32 weeks depending upon the time of diagnosis of gestational diabetes and for pre-diabetic women, fetal echocardiography was done at 24-26 weeks of gestation. Statistical analysis was done using the Statistical Package of the Social Sciences version 20.

**Results:**

Among 104 patients, 16 (15.38%) patients had abnormal fetal echocardiography. Eighty-three (79.81%) were gestational diabetics, 21 (20.19%) were pre-existing diabetic women. Among 83 gestational diabetes, 7 (8.4%) had abnormal echo finding and among 21 pre-existing diabetics, 9 (42.8%) had abnormal echo finding.

**Conclusions:**

There was an increased chance of fetal cardiac malformation in gestational diabetic and pre-existing diabetics diabetic especially in an uncontrolled glycemic state. And, if they were diagnosed prenatally, clinical outcomes for both mother and fetus would have been better.

## INTRODUCTION

Gestational diabetes has been a common condition in pregnancy nowadays, with a prevalence of 1.56% in our population. The incidence of congenital malformation among newborn in maternal diabetics than that of the normal population has increased by five times.^1–3^ Cardiac malformation is one of the most common types of malformation which occurs in 8.5% of the cases.^3–5^ Common malformations seen in fetuses are VSD, the transformation of great vessels, aortic stenosis, pulmonary atresia, dextrocardia and conotruncal defects.^6–8^

Despite good glycemic control, hyperinsulinism and fetal hyperglycemia can cause hypertrophic cardiomyopathy which is the most common congenital abnormality of heart in the infants of diabetic mothers and is found in 30% of the cases, sometimes leading to sudden intrauterine fetal death.^9^

The objective of the study is to find out the prevalence of abnormal fetal echocardiography in pre and gestational diabetes in pregnant women attending Kathmandu Medical College Teaching Hospital.

## METHODS

This is a descriptive cross-sectional study conducted from April 15, 2017, to April 14 2018 in Kathmandu Medical College Teaching Hospital. Ethical approval was obtained from the institutional review committee of Kathmandu Medical College before the initiation of the study. The convenient sampling method was used. The pregnant women who were attending Kathmandu Medical College Teaching Hospital and once diagnosed as gestational diabetic and pre-existing diabetic, they were advised for fetal echocardiography and included in the study. Informed consent was taken from all the participants. Detail clinical history was taken and patients with heart disease, family history of congenital heart disease, hypertensive disorder, history of exposure to cardiac teratogens were excluded from the study.

Sample size calculation,

$$\begin{array}{ll} \text{n} & \text{= }{\text{Z}}^{\text{2}}\times \text{p}\times \text{q}/{\text{e}}^{\text{2}}\\ & \text{= }{\left(1.96\right)}^{\text{2}}\times 0.5\times (1-0.5)/{(\text{0.1)}}^{\text{2}}\\ & \text{= }96 \end{array}$$

Where,

n = Sample size
Z = 1.96 at 95% Confidence Interval
p = 0.5
q = 1-p
e = Margin of error, 10%

The required sample size was 96. However, the total sample size of 104 was taken.

Gestational diabetes was confirmed by a 75-gram oral glucose tolerance test in late second trimester and third trimester and per gestational diabetes by medical history. Fetal echocardiography was mainly done at a gestational age of 22-32 weeks depending upon the time of diagnosis of gestational diabetes and for pre-diabetic women, fetal echocardiography was done at 24-26 weeks of gestation. All prediabetics were diagnosed on the first antenatal visit from medical history, usages of medication, and their sugar level. Gestational diabetes screening was done at 24 to 28 weeks and few were diagnosed later which might manifest other symptoms. Statistical analysis was done using the Statistical Package of the Social Sciences version 20.

## RESULTS

Among 104 patients, 16 (15.38%) patients had abnormal fetal echocardiography which was done in the second trimester and early third trimester. In our study out of 104, 83 (79.81%) had gestational diabetes and 21 (20.19%) had preexisting diabetes. Among 83 gestational diabetes, seven (8.4%) had abnormal findings in fetal echocardiography. Out of which four (4.8%) had small echogenic foci in ventricles, two (2.4%) had ventricular septal defects and one (1.2%) had atrial septal defects. In the pre-existing diabetes group, 9 (42.8%) had abnormal findings in fetal echocardiography. Three had multiple defects like Tetralogy of Fallot, single ventricle and tricuspid atresia, two had a small ventricular septal defect, two had small foci in ventricles, one had pulmonary atresia and one had dextrocardia. (Table 1).

**Table 1:** Abnormal finding in fetal echocardiography in gestational diabetics.

Abnormal findings	n (%)
Small echogenic foci in ventricles	4 (4.8)
Ventricular septal defects	2 (2.4)
Atrial septal defects	1 (1.2)

In the pre-gestational diabetes group, nine (42.8%) had abnormal fetal echocardiography findings. Three (14.3%) had multiple defects and could be fatal in later life so termination was requested. Two (9.6%) had a ventricular septal defect, two (9.6%) had small echogenic foci in ventricles, one (4.8%) had dextrocardia, one (4.8%) had pulmonary atresia (Table 2).

**Table 2:** Abnormal finding in fetal echocardiography in pre-existing diabetics.

Abnormalities	n (%)
Multiple defects like tetralogy of Fallot, single ventricle, tricuspid atresia	3 (14.3)
Small ventricular septal defect	2 (9.6)
Small echogenic foci in ventricles	2 (9.6)
Pulmonary atresia	1 (4.8)
Dextrocardia	1 (4.8)

Out of 83 echocardiography in gestational diabetes women, seven (8.4%) had abnormal echocardiography reports. Whereas out of 21 pregestational diabetic women, nine (42.8%) echocardiography reports were abnormal. Among nine, three (33.33%) had multiple cardiac deformities which could be fatal therefore requested for termination of pregnancy at 22 weeks of gestation.

## DISCUSSION

A small rise in circulating blood glucose levels may be hazardous to the developing embryo and good glycemic levels have shown to reduce the rate of congenital abnormalities, preterm delivery, and stillbirths.^10,11^ In normal pregnancies there is an increase in maternal insulin resistance due to maternal physiological adaptation which occurs to ensure adequate fetal growth and development. Pregnancy hormones and other factors are thought to interfere with the action of insulin as it binds to the insulin receptor.^12^ Lisowski et al.^1^ reported there is 10 times increased in the incidence of cardiac malformation among newborns in maternal diabetics than that of the normal population but our study showed much higher 8.4% in gestational diabetes and 42.8% in pregestational diabetes. Kozak-Barany A et al.^6^ had found VSD, the transformation of great vessels, aortic stenosis, pulmonary atresia, dextrocardia and conotruncal defects in their echocardiography and in our study which was more similar echocardiography finding tetralogy of Fallot, single ventricle, tricuspid atresia, ventricular septal defect, atrial septal defect, small echogenic foci in ventricles, pulmonary atresia and dextrocardia. Montserrat Balsells et al.^9^ in their review they have noted hypertrophic cardiomyopathy which sometimes leads to sudden intrauterine fetal death but in our study, there was not any hypertrophic cardiomyopathy finding in echocardiography and any intrauterine fetal death.

## CONCLUSIONS

There are very few studies done about fetal echocardiography in our context and even fewer fetal echocardiography in pregestational and gestational diabetes so it is very difficult to conclude the definite pattern or abnormalities of the fetal heart seen in these patients. Fetal echocardiography of abnormal reports should be followed until delivery and repeated echocardiography should be done to clarify the condition and even follow up to early childhood condition. There are many treatable conditions after delivery or even in utero, so knowing about the condition beforehand will certainly help both parents and doctors to manage the condition.

## Conflict of Interest

**None.**
